# Clinical Application of In Vitro Tests for COVID-19 Vaccine Delayed Hypersensitivity Diagnostics

**DOI:** 10.3390/ijms241713296

**Published:** 2023-08-27

**Authors:** Jan Romantowski, Aleksandra Górska, Maciej Zieliński, Piotr Trzonkowski, Karolina Rucka, Marek Niedoszytko

**Affiliations:** 1 Department of Allergology, Medical University of Gdansk, 80-414 Gdańsk, Poland; aleksandra.gorska@gumed.edu.pl (A.G.); mnied@gumed.edu.pl (M.N.); 2 Department of Medical Immunology, Medical University of Gdansk, 80-414 Gdańsk, Poland; maciej.zielinski@gumed.edu.pl (M.Z.); ptrzon@gumed.edu.pl (P.T.); krucka@uck.gda.pl (K.R.)

**Keywords:** delayed hypersensitivity, COVID-19, vaccine, allergy, cytometry

## Abstract

Drug hypersensitivity reactions can be classified as immediate or delayed. While diagnostic options for immediate reactions are well developed and standardized, delayed reactions (in many cases type IV according to Gell and Coombs) are a challenge for allergy work-up. In recent years, some in vitro markers have been proposed and used for delayed reactions, such as contact dermatitis. Primary strategy: Avoidance is difficult to achieve, especially for COVID-19 vaccinations, when immunity against infection is extremely important. The aim of our study was to evaluate the application of in vitro delayed hypersensitivity tests in COVID-19 vaccines. Seven patients with a positive history of severe delayed drug allergy were enrolled. Vein blood was collected to stimulate cells with the tested vaccines (Comirnaty, Janssen, Spikevax) and excipients with the assessment of CD40L, CD69, IL-2, IL-4, IL-6, IL-10, IFNgamma, TNFalfa, and intracellular markers: granulysin and INFgamma. In addition, basophile activation tests, patch tests, skin prick tests, and intradermal tests were performed with the tested vaccine. Finally, the decision was made to either administer a vaccine or resign. Two out of seven patients were considered positive for drug hypersensitivity in the in vitro test according to the high vaccine stimulation index measured with CD69 (6.91 and 12.18) and CD40L (5.38 and 15.91). All patch tests, BATs, and skin tests were negative. Serum interleukin measurements were inconclusive as the impact of the vaccine itself on the immunity system was high. Intracellular markers gave uncertain results due to the lack of stimulation on the positive control. CD69 and CD40L could be reliable in vitro markers for delayed hypersensitivity to COVID-19 vaccines. Patch tests, skin tests, BATs, and serum interleukins did not confirm their usefulness in our study.

## 1. Introduction

Drug hypersensitivity reactions can be classified as immediate (usually <1 h to symptom onset) and delayed (usually >1 h; though multiple reactions occur after 24 h) [1]. Typical symptoms of immediate reactions are urticaria, anaphylaxis, rhinitis, bronchi constriction, and edema. These result from either the immunological Type 1 IgE-related release of histamine, prostaglandins, tryptase, platelet activating factor, etc., or non-allergic hypersensitivity (for example, NSAIDS or radiologic contrast dyes) that also results in an influx of histamine, leukotrienes, or complement activation [2]. Type 2 and Type 3 immune reactions are rare, while delayed hypersensitivity reactions are usually Type 4 and mediated by sensitized T cells [1,2]. The symptoms vary greatly and include skin manifestations, e.g., contact dermatitis, fixed drug eruption, acute, generalized exanthematic pustulosis, maculopapular rash, drug reaction with eosinophilia, and systemic symptoms, e.g., Lyell’s and Stevens–Johnson Syndrome, and Toxic Epidermal Necrolysis (TEN), and also granulocytopenia, renal, and liver dysfunctions [1].

Delayed hypersensitivity reactions may also occur after COVID-19 vaccination. In terms of allergic reactions, these vaccines are considered relatively safe in the current literature [3]. Xu et al. analyzed 20,657 doses of Comirnaty and Spikevax vaccinations and detected only 138 reports of allergy-like reactions (0.006%) [4]. Out of these, 109 were immediate and, after thorough evaluations and exclusion of known side effects such as fever, only 10 were described as delayed allergic reactions. The symptom onset time varied from 8 h to 7 days from vaccine administration. Symptoms included edema, urticarial rash, large persisting local reactions, and a single case of serum sickness disease. Out of these patients, only 60% had any allergic history. With the general allergy prevalence estimated at 40–50% by the World Health Organization, it might be difficult to predict such reactions based only on patients’ medical history [5].

The diagnostic methods for immediate reactions, which are well developed and validated, include skin prick tests (SPTs), intradermal tests (IDTs), serum allergen-specific IgE, and basophil activation tests (BATs), along with oral challenges and provocations [6]. In delayed reactions, most of these tests are hardly useful. Patch tests and IDTs with late read are used, although their usefulness is still uncertain. Provocations are difficult to perform due to the required long observation period, as a reaction may occur even after several days. Provoking the patient with a suspected excipient might also be hazardous, especially in those with a severe delayed reaction history, such as TEN. In the EAACI 2022 position paper by Barbaud et al., in patients with delayed reactions after COVID-19 vaccine administration, excipient skin tests are not recommended, although premedication with antihistamines might be discussed. The recommendations suggest that in the case of a delayed hypersensitivity reaction (>4 h) history, then a long observation period of at least 3 h should be applied [7].

The uncertainty of delayed hypersensitivity reaction diagnosis includes possible in vitro tests. The Lymphocyte Transformation Test (LTT) was developed to measure 3H-thymidine during DNA synthesis after 6 days of culture T cells with the tested drug [8]. Other tests include measurement markers of T-cell activation (CD69, CD25), cytokine levels (IL-2, IL-5, IFNgamma, TNF-alfa), and proliferation markers post PBMC allergen exposure in vitro [9]. Studies suggest the role of granulysin in early Stevens–Johnson Syndrome [8,10]. The main function of the CD40/CD40L system is the co-stimulation of B cells, although CD40L is soluble in serum and acts as a cytokine [11,12]. It activates B cells but also monocytes, thus enhancing the proinflammatory response. In studies in murine models, blocking the CD40 ligand (CD40L) induced some tolerance in delayed contact hypersensitivity [13].

The aim of this study was to evaluate a COVID-19 vaccine allergy using in vitro T-cell exposure to the tested drug with the flow cytometry measurement of CD69, CD40L, TNFa, IFNG, IL-2, IL-4, IL-6, IL-10, intracellular granulysin, and IFNgamma. Additionally, in vivo tests were performed: skin prick tests (SPTs), intradermal tests (IDTs), and patch tests. The aim was to finally either continue or halt vaccination in patients who experienced late hypersensitivity drug reactions.

## 2. Results

A total of seven patients were tested with COVID-19 vaccines: Janssen (3), Comirnaty (3), and Spikevax (1) (Table 1). The decision was made together with the patients to avoid previously used vaccines or excipients. The patients’ characteristics, patch test results, and final drug administration are included in Table 1.

The results of the stimulation index for BATs, CD69, and CD40L are presented in Table 2. A strong Pearson’s correlation (r = 0.96) was detected between CD69 and CD40L. The stimulation index for Th1/Th2 cytokines is presented in Table 3. The results for the stimulation index of the intracellular markers granulysin and IFN-gamma are presented in Table 4. According to Table 1, five out of the seven patients were considered negative during the in vitro tests and patch tests. In these cases, the SPT and IDT were performed with negative results in all cases. In five cases, the diagnostic process was followed by vaccination using the tested vaccine (Table 1). All vaccinations were performed without clinically significant side effects with no skin adverse reactions. Two patients were evaluated as hypersensitive according to the results of the CD69 and CD40L stimulation index and an SPT, IDT, and vaccination were not performed. In these cases, intracellular markers were also positive, though this assessment showed uncertain results (Table 4)

The two patients who were considered allergic according to CD69/CD40L showed different cytokine indices compared to the control group and negative patients: (1) The mean index was higher for IL-2 (22,581 vs. 15,212), IL-4 (22,523 vs. 14,010), and IL-10 (44,319 vs. 12,557); and (2) the mean index was lower for TNF-alfa (11,707 vs. 19,601), IFN-gamma (4848 vs. 114,031), and IL-6 (131 vs. 8959). However, due to the high number of outlier values in this small sample, no statistic test was performed.

## 3. Discussion

Our study addressed an issue presented in the EAACI 2022 position paper by Barbaud et al. [7] with seven patients that could not be vaccinated in standard medical units due to either hesitancy to get vaccinated or medical disqualification. The reason was usually a fear of facing a severe delayed hypersensitivity reaction for patients with Lyell’s or Stevens–Johnson Syndrome. It is difficult to observe such patients post-vaccination for a long time, and the standard protocols for safe vaccination do not include prolonged patient stays in medical facilities [3]. In such cases, most patients decline vaccination. This decision is understandable considering the severity of past drug-related reactions and lack of diagnostic options, though there is little medical basis for such fears.

**Figure 1:**
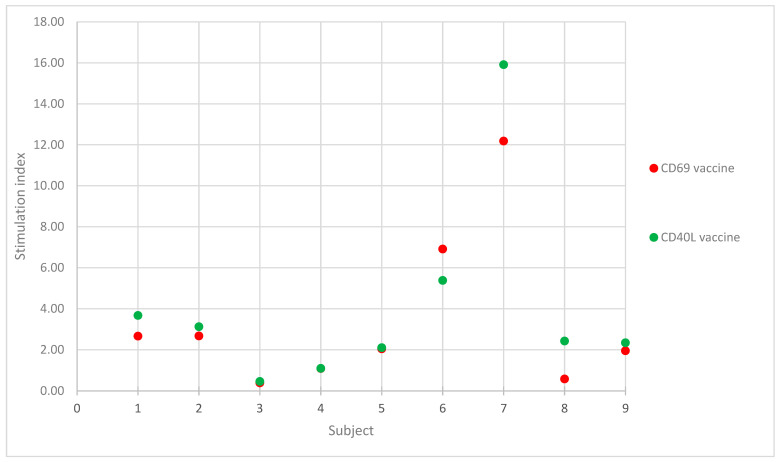
Graphical presentation of tested vaccine stimulation index of CD69 and CD40L for 7 patients and 2 healthy controls. X axis represents patients. Patients 1–5 had negative results. Patients 6 and 7 had positive results. Patients 8 and 9 were healthy controls with negative results.

Based on the results of our seven patients and two healthy controls (Figure 1), the CD40L and CD69 markers detected hypersensitivities based on lymph cell activation. Furthermore, both markers correlated strongly with each other. In the current literature, CD69 is considered superior to other activation markers, such as CD25 or CD71, due to its large difference from the baseline values and fast (24 h) results [8,14,15,16]. Considering the fact that delayed hypersensitivity reactions usually start after 24 h, the timing of the CD69 marker is clinically satisfactory [17]. Our study is in line with those studies, as shown in Table 2 and Figure 1**,** showing a clear difference between the negative results, healthy controls, and positive results. Beeler et al. assessed CD69 after 48 h [16]; however, other studies, including ours, showed that 24 h is also valid [18]. CD40L activation in delayed hypersensitivity is a novel concept as its main function is co-stimulation of B cells. According to Takeuchi et al., CD40L is associated not only with Th2 cytokines such as IFNG but also with Th1 cytokines such as IL-10 [19]. Tang et al. revealed that it plays an important role in contact dermatitis [13]. The authors suggested that this effect is due to the blocking of IL-12. Deng et al. showed that CD40L is key in the maturation of dendritic cells in the development of asthma, and with this mechanism, delayed hypersensitivities might also be affected [20].

The classic LTT was not included in the diagnostic protocol, as it takes 6 days to perform and the decision to either vaccinate a patient or not was supposed to be made within the 5 days of a standard work week [8]. In this case, the time required for the clinical decision process is important as patients reluctant to receive a vaccine tend to be more anxious the longer they wait [3,7].

The BAT did not reveal any correlation with CD40L/CD69. In one case, despite a positive result in PEG stimulation (patient 4; Table 2), vaccination was performed with no adverse effects. The BAT is promising in multiple other clinical indications, such as insect venom or food allergy, particularly in cases where the specific IgE and SPT are non-conclusive in the allergy work-up [21]. Its usefulness is highlighted in patients with mast cell diseases such as mastocytosis that might have low concentrations of IgE and still suffer from immediate allergic reactions [22]. However, for drug hypersensitivity, its sensitivity is estimated at 55% and its specificity at 80%. The main utility of the BAT remains immediate reactions, though successful application in delayed reactions has been reported, for example, in radiologic contrasts and beta-lactams [23,24]. In our opinion, our study supports the superiority of CD40L/CD69 over the BAT in COVID-19 vaccine hypersensitivities during delayed reactions.

Cytokine concentration (TNF-alfa, IFN-gamma, IL-2, IL-4, IL-6, and IL-10) increased greatly after in vitro exposure to the COVID-19 vaccine. Cytokine synthesis is considered sensitive, although its specificity is low [8]. Th1 cytokines TNF-alfa, IFN-gamma, and IL-2 are more useful in delayed drug reactions, while IL-4 and IL-10 would represent immediate reactions [25,26]. In our study, we observed an extreme rise in TNF-alfa and IFN-gamma, as well as a rise in all the other cytokines (Table 3) upon in vitro exposure to the COVID-19 vaccine. This increase was also present in healthy controls, and in all the vaccinated patients (subjects 1–5 and the controls), it was false-positive. This is in line with other studies that highlight the low specificity of cytokines in the delayed drug hypersensitivity diagnostic process [8,25,26]. In our opinion, it might be more evident in vaccines that activate the immune system as their primary mechanism. A rise in the number of these cytokines has been observed post-vaccination when using such vaccines as influenza and COVID-19 and might be more evident in subjects who have already had contact with either virus or their first dose of a vaccine [27,28]. With the ongoing high COVID-19 transmission in 2022, it was difficult to evaluate individuals’ exposure [29]. This would suggest that the utility of Th1/Th2 cytokines in vaccine allergy diagnostics is highly limited.

In our study, intracellular granulysin and IFN-gamma revealed unreliable results due to the lack of a positive control response in the samples. Still, as presented in Table 4, patients 6 and 7 presented higher results of the stimulation index for vaccine provocation than the healthy control (C1). These were the same patients identified as allergic in CD69/CD40L identification, so this result is in line with our primary result, though the number of satisfactory samples was too low to draw any conclusions. These markers might address more mechanisms than CD69, the BAT, or cytokines [8]. They might be a significant addition as they measure the effector cell cytotoxic functions that are particularly present in severe delayed reactions, such as Stevens–Johnson Syndrome. Currently, more studies in the future are required to validate this method.

The question of whether to perform tests with excipients or vaccines remains open. Barbaud et al. [7] suggests that in cases of a severe drug-related reaction history, excipients should be investigated (PEG, polysorbat, etc.). In the case of testing patients who experienced an allergic reaction to the first dose of a vaccine, a complete drug should be used. Romantowski et al. performed in vivo tests on a large group of patients with vaccines, not excipients, with satisfactory results [3]. In our study, with the exception of the BAT, PEG stimulation did not show sensitization responses from cells. Patient 4, with a positive BAT result, was vaccinated with Vaxzevria, which does not include PEG. In patient number 7, positive results were acquired with Comirnaty and negative results with PEG. Since Comirnaty contains PEG, there should be another source of sensitization. This supports the opinion that testing with PEG might not be sufficient prior to vaccination. Also, other studies suggest that testing with PEG might provide unreliable results, and patients with positive tests still undergo vaccination with no adverse events [30,31]. Still, excipient tests might provide patients vital information on using other drugs in the future as they usually contain similar substances.

Finally, our study revealed that all the in vivo tests, i.e., the SPTs, IDTs, and patch tests, did not show any positive results. Patch tests’ main utility is in contact dermatitis diagnosis, in which they are considered the golden standard in the detection of allergies to haptens such as nickel or paraphenyldiamine [32]. Only a few drugs for this purpose, such as NSAIDs, anticonvulsants, and glicocorticosteroids, are standardized and commercially available. Other drugs need to be prepared in-house from intravenous drugs with dilution 10% in petrolatum. A lack of standardization results in either false negatives when the concentration is too low or false positives when the irritative skin effect of the drug appears [32]. SPTs and IDTs are used to detect allergen-specific IgE in the skin and are useful in immediate allergic reactions, especially in the case of airborne, food, or insect allergies [33]. However, many drugs generate non-IgE-dependent reactions [34,35]. In these cases, these tests’ sensitivity is low. Still, these tests might be used to investigate immediate reactions to COVID-19 vaccination, as investigated by Romantowski et al. [3].

This supports the general opinion that skin tests and patch tests have a very limited and controversial place in allergy work for delayed hypersensitivities. In our opinion, in vivo tests results might be less reliable considering delayed vaccine hypersensitivity and create a greater burden for the patient (for example, risk of anaphylaxis, contact dermatitis exacerbation, and antihistamine withdrawal in some cases).

The weak point of this study is the sample size, which included seven patients and two healthy controls. Validation of the test for clinical use requires a larger patient group. However, enrolling large number of such patients might be difficult even in multicenter studies due to (1) the rarity of delayed hypersensitivity reactions post COVID-19 vaccinations; (2) the heterogeneity of groups of patients with very different diagnoses, such as Stevens–Johnson Syndrome vs. a large local reaction; (3) the reluctance of many individuals to be vaccinated [3,4,7] In addition, in drug hypersensitivity, the golden standard is allergen provocation, and its application would exclude definitely false-positive results. However, it is considered contraindicated when patients’ symptom history includes severe life-threatening reactions such as anaphylactic shock or Stevens–Johnson Syndrome. In this clinical circumstance, the benefits of provocation results are lower than the risk of a reaction.

The main strength of this study is the clinical outcome. Five out of seven patients who would not have had a chance to receive a COVID-19 vaccine were successfully vaccinated. These real-life results provide an option for practicing physicians and address the issue highlighted by the EAACI position paper [7]. It also presents the superiority of CD69 and CD40L markers over cytokine concentration, BATs, and in vivo tests in COVID-19 vaccine allergy testing, which might decrease the cost of allergy diagnostics in the future.

## 4. Materials and Methods

### 4.1. Study Design

A total of 7 patients were enrolled in the study and signed written informed consents. Inclusion criteria included: aged over 18 and drug-related severe delayed hypersensitivity history (such as Lyell, TEN, Stevens–Johnson Syndrome, severe drug-induced rash, serum sickness disease) that led to disqualification from COVID-19 vaccination. Exclusion criteria included: no consent for vaccination, pregnancy, exacerbated chronic disease, and acute infectious disease. Allergy history and comorbidities were collected for all patients. Patch tests were performed with the Polish Baseline Series containing 25 contact haptens, and additionally, a patch test was carried out with the investigated drug (diluted; 1:10 in petrolatum) [36]. Patch tests were evaluated at the 48 and 72 h timepoints. T cells were isolated from peripheral blood. A culture was exposed in vitro to the investigated drug, with a positive and negative control. A BAT was performed with the investigated drug. The BAT result was obtained 6 h after in vitro exposure to the tested vaccine. After 24 h, the measurement of CD69 and CD40L was completed. At 48 h, the Th1/Th2 cytokines TNF-alfa, IFN-gamma, IL-2, IL-4, IL-6, and IL-10 were measured. Finally, at the 72 h timepoint, intracellular granulysin and IFN-gamma were measured. In the case of negative results, the patients proceeded with vaccine SPTs (undiluted vaccine; cut-off point: 3 mm wheal) and IDTs (concentrations: 1:100 and 1:10; cut-off point: increase in primary wheal by 3 mm), with readings after 15 min [3,33]. After all tests were concluded, a decision was made on the tested vaccine administration. The cut-off point for the stimulation index for CD69 and CD40L was set at 4 as a moderate activation, according to previous studies on delayed hypersensitivity [16,37]. The cut-off point for the BAT was set at 2.5 according to previous studies [38]. The study was approved by the Bioethics Commission in Medical University of Gdansk.

Final data were analyzed using the Statistica 13 computer program. The relationship between particular tests was analyzed using Pearson’s correlation.

### 4.2. Flow Cytometry

The BAT was performed strictly according to the manufacturer’s procedure (BÜHLMANN Laboratories AG, Schönenbuch, Switzerland). Briefly, 50 μL of EDTA whole blood was stimulated with the COVID-19 vaccine at neat and diluted concentrations at 37 °C and stained with anti-CCR3 and anti-CD63 fluorochrome-labeled antibodies. As a positive control, fMLP (N-Formylmethionyl-leucyl-phenylalanine) was used, and anti-FceRI antibodies were used as a negative control and PBS buffer. Next, the samples were lysed, washed, and read out using a Navios flow cytometer (Beckman Coulter, Brea, CA). Basophils were gated as SSClow/CCR3+, and the CD63 expression was measured. The results were calculated according to the background CD63 staining (PBS buffer) and as an index (stimulated/unstimulated sample). As the patient’s result, the highest index was taken from the neat and diluted stimuli.

Next, PBMC heparin whole blood was obtained via density centrifugation (Ficoll-Paque PLUS, GE HealthCare, Chicago, IL, USA), and 100,000 cells were seeded per well for stimulation. The cells were stimulated with the COVID-19 vaccine at neat and diluted concentrations at 37 °C/5% CO_2_/RPMI 1640—Cell Culture Media for 24 h (CD69/CD40L testing), 48 h (cytokines testing), or 78 h (granulysin and IFN gamma). RPMI was used as a negative control, and a cell stimulation cocktail (phorbol 12-myristate 13-acetate and ionomycin, ThermoFisher Scientific, Waltham, MA, USA) was used as a positive control. The expression of CD69/CD40L was assessed upon staining with fluorescent-conjugated antibodies: CD45 (KC56 (T-200) clone), CD3 (UCHT1 clone), CD8 (B9.11 clone), CD69 (TP1.55.3 clone), and CD40L (TRAP-1 clone). The cells were gated first as SSclow/CD45+, and then CD3+/CD8- (CD4-positive T cells) and singlets (SSc A/H), and finally, the percentages of CD40L and CD69 were measured against fluorescence minus one control. As the patient’s result, an index (stimulated/unstimulated sample) was calculated, and the highest index was taken from the neat and diluted stimuli. The Navios flow cytometer (Beckman Coulter, Brea, CA, USA) was used for the sample readout. The minimum cell acquisition number per sample was 50,000 events.

The Th1/Th2 cytokines were tested with a Human Th1/Th2 Cytokine Cytometric Bead Array-CBA (BD Bioscience, Franklin Lakes, NJ, USA), strictly according to the manufacturer’s procedure. After 48 h of cell stimulation, the supernatant was taken out and tested with a CBA assay. As the patient’s result, an index (stimulated/unstimulated sample) was calculated, and the highest index was taken from the neat and diluted stimuli. The BD FACSVia flow cytometer (BD Bioscience, Franklin Lakes, NJ, USA) was used for the sample readout.

Finally, the granulysin and IFN gamma was tested after 78 h of stimulation. Before testing, the cells were treated with a Protein Transport Inhibitor Cocktail (Brefeldin A and Monensin, ThermoFisher Scientific Waltham, MA, USA) and then stained with fluorescent-conjugated antibodies: CD45 (KC56 (T-200) clone), CD3 (UCHT1 clone), CD8 (B9.11 clone), CD56 (N901 (NKH-1) clone), IFN gamma (45.15 clones), and granulysin (eBioDH2 clone). The cells were gated first as SSclow/CD45+, and then CD3+/CD8+ (or CD3-/CD56+) and singlets (SSc A/H), and finally, the percentages of IFN gamma and granulysin were measured against fluorescence minus one control for both cytotoxic T cells (CD3+/CD8+) and NK cells (CD3-/CD56+). For the patient’s result, an index (stimulated/unstimulated sample) was calculated, and the highest index was taken from the neat and diluted stimuli. The Navios flow cytometer (Beckman Coulter, Brea, CA, USA) was used for the sample readout. The minimum cell acquisition number per sample was 50,000 events.

## 5. Conclusions

Our study suggests CD69 and CD40L are vital in the diagnosis of COVID-19 vaccine delayed hypersensitivity and could be included in allergy work prior to vaccination. Our study did not confirm the utility of SPTs, IDTs, patch tests, TNF-alfa, IFN-gamma, IL-2, IL-4, IL-6, IL-10, granulysin, or intracellular IFN-gamma in this clinical situation. We present a possible clinical management of suspected delayed hypersensitivity for COVID-19 vaccines that would enable vaccination in test-negative subjects.

## Figures and Tables

**Table 1:** Clinical characteristics and results of patch tests, skin prick tests and intradermal tests. NSAIDs—Non-Steroid Anti-inflammatory Drugs; TEN—Toxic Epidermal Necrolysis; n/d—not done; Neg—negative; F—female; M—male.

Patient Number	Allergic Reaction in the Past	Allergen in the Past Causing Delayed Allergic Reactions	Age	Gender	Vaccine that Was Used for All Tests	Patch Tests Standard Allergens	Patch Tests—Investigated Vaccine/Drug	Skin Prick Tests and Intradermal Tests	Vaccine/Drug Administration
1	Lyell	Lamotrigine	21	F	Janssen	Neg	Neg	Neg	Yes
2	Lyell	NSAIDs, paracetamol, sumatriptan	40	F	Janssen	Neg	Neg	Neg	Yes
3	Stevens–Johnson	Topiramate	19	M	Comirnaty	Neg	Neg	Neg	Yes
4	Drug-induced rash	Vaxzevria	40	F	Comirnaty	Formaldehyde, fragrance mix, palladium, linalyl, limonene, methylisothiazolinone, textile dyes	Neg	Neg	Yes
5	Drug-induced rash	Comirnaty	39	F	Spikevax	Neg	Neg	Neg	Yes
6	TEN	Comirnaty	47	F	Janssen	Nickel, cobalt, Peru balsam	Neg	n/d	No
7	TEN	NSAIDs	35	M	Comirnaty	Neg	Neg	n/d	No

**Table 2:** Results of stimulation index for 7 patients and 2 healthy controls together with final interpretation by the authors. Bold font represents results that crossed the cut-off threshold (2.5 for BAT and 4.0 for CD40L and CD69). BAT—basophil activation test; n/d—not done; C—healthy control.

Patient Number	BAT Index with Tested Vaccine	BAT Index with PEG	CD69 Index with Tested Vaccine	CD69 Index with PEG	CD40L Index with Tested Vaccine	CD40L Index with PEG	Proposed Interpretation for the Decision on Vaccine Administration
1	0.19	0.60	2.66	0.55	3.67	0.73	Negative
2	0.13	0.80	2.67	0.61	3.12	0.59	Negative
3	0.13	2.14	0.39	0.37	0.46	0.49	Negative
4	1.00	**3.80**	1.09	1.27	1.10	1.27	Uncertain
5	0.60	1.23	2.04	1.41	2.10	1.32	Negative
6	0.12	0.51	**6.91**	1.77	**5.38**	0.65	Positive
7	0.36	**3.57**	**12.18**	1.71	**15.91**	1.37	Positive
C1	1.05	0.51	0.58	n/d	2.42	n/d	Negative
C2	0.71	0.48	1.95	n/d	2.34	n/d	Negative

**Table 3:** Stimulation index of selected Th1/Th2 cytokines after 48 h of incubation of patients’ serum together with either tested vaccine or PEG.

Patient Number	TNF-Alfa Index with Tested Vaccine	TNF-Alfa Index with PEG	IFN-Gamma Index with Tested Vaccine	IFN-Gamma Index with PEG	IL-2 Index with Tested Vaccine	IL-2 Index with PEG	IL-4 Index with Tested Vaccine	IL-4 Index with PEG	IL-6 Index with Tested Vaccine	IL-6 Index with PEG	IL-10 Index with Tested Vaccine	IL-10 Index with PEG
1	34,626.27	24.40	476,464.00	1.20	17.67	0.97	3.60	0.56	49.64	3.01	9.53	2.50
2	33,485.00	0.00	55,715.00	0.00	2.12	0.21	16.29	4.43	229.23	5.37	1.32	2.60
3	15.85	524.00	513.00	0.00	10.18	2.06	6.70	1.43	7.78	1.73	11.30	1.95
4	77.11	39.14	167.86	8.71	21.31	4.11	4.97	2.71	9.78	2.75	14.07	3.30
5	306.04	0.00	0.00	0.00	8.00	4.00	0.00	0.00	4930.40	66.41	53.21	1.29
6	23,213.10	0.06	8879.03	0.06	3.03	1.43	3.70	2.69	137.80	0.03	3.08	2.28
7	201.46	0.84	818.69	0.01	175.10	1.02	3.53	0.81	124.61	1.56	2.19	1.23
C1	27.74	6.27	2055.27	1.09	3.48	1.38	5.40	1.37	44.22	3.86	19.21	3.12
C2	68,675.00	0.00	263,306.29	11.71	214.17	2.56	107.00	60.00	59.97	0.49	153.61	1.77

**Table 4:** Results of stimulation index for intracellular markers: granulysin and IFN-gamma.

Patient Number	Intracellular Granulysin Index with Tested Vaccine	Intracellular Granulysin Index with PEG	Intracellular IFN-Gamma Index with Tested Vaccine	Intracellular IFN-Gamma Index with PEG	Comments
1	28.93	69.53	n/d	n/d	Unreliable due to lack of stimulation in the positive control for intracellular IFN-gamma
2	4.76	8.26	n/d	n/d	Unreliable due to lack of stimulation in the positive control for intracellular IFN-gamma
3	n/d	n/d	n/d	n/d	Unreliable due to lack of stimulation in the positive control for both tests
4	n/d	n/d	n/d	n/d	Unreliable due to lack of stimulation in the positive control for both tests
5	n/d	n/d	n/d	n/d	Unreliable due to lack of stimulation in the positive control for both tests
6	12.47	2.39	8.28	8.81	
7	4.57	0.42	1.04	2.52	
C1	0.68	n/d	0.39	n/d	
C2	n/d	n/d	n/d	n/d	Unreliable due to lack of stimulation in the positive control for both tests

## Data Availability

The data presented in this study are available on request from the corresponding author.

## References

[1] Böhm R., Proksch E., Schwarz T., Cascorbi I. (2018). Drug Hypersensitivity: Diagnosis, Genetics, and Prevention. Dtsch. Ärzteblatt Int..

[2] Rra C. (1968). Coombs Classification of Allergic Reactions Responsible for Clinical Hypersensitivity and Disease. Clinical Aspects of Immunology.

[3] Romantowski J., Kruszewski J., Solarski O., Bant A., Chciałowski A., Pietrzyk I., Sańpruch P., Górska A., Chełmińska M., Knurowska A. (2022). Protocol of Safe Vaccination against COVID-19 in Patients with High Risk of Allergic Reactions. Clin. Transl. Allergy.

[4] Xu J., Vanijcharoenkarn K., Sexton M.E., Martin L., Lee F.E.H., Kuruvilla M.E. (2021). Delayed Hypersensitivity Reactions Following First Dose of the SARS-CoV2 MRNA Vaccines. J. Gen. Intern. Med..

[5] Pawankar R., Canonica G.W., Lockey R.F., Holgate S.T. (2011). White Book on Allergy 2011–2012: Executive Summary.

[6] Brockow K., Garvey L.H., Aberer W., Atanaskovic-Markovic M., Barbaud A., Bilo M.B., Bircher A., Blanca M., Bonadonna B., Campi P. (2013). Skin Test Concentrations for Systemically Administered Drugs—An ENDA/EAACI Drug Allergy Interest Group Position Paper. Allergy Eur. J. Allergy Clin. Immunol..

[7] Barbaud A., Garvey L.H., Arcolaci A., Brockow K., Mori F., Mayorga C., Bonadonna P., Atanaskovic-Markovic M., Moral L., Zanoni G. (2022). Allergies and COVID-19 Vaccines: An ENDA/EAACI Position Paper. Allergy.

[8] Porebski G., Gschwend-Zawodniak A., Pichler W.J. (2011). In Vitro Diagnosis of T Cell-Mediated Drug Allergy. Clin. Exp. Allergy.

[9] Sachs B., Fatangare A., Sickmann A., Glässner A. (2021). Lymphocyte Transformation Test: History and Current Approaches. J. Immunol. Methods.

[10] Abe R., Yoshioka N., Murata J., Fujita Y., Shimizu H. (2009). Granulysin as a Marker for Early Diagnosis of the Stevens–Johnson Syndrome. Ann. Intern. Med..

[11] Fitzgerald K.A., O’Neill L.A.J., Gearing A.J.H., Callard R.E. (2001). CD40L. The Cytokine FactsBook and Webfacts.

[12] Jasińska J. (2015). Rola Układu Receptor CD40—Ligand CD40 (CD40/D40L) w Procesach Zapalnych. Alergia.

[13] Tang A., Judge T.A., Turka L.A. (1997). Blockade of CD40-CD40 Ligand Pathway Induces Tolerance in Murine Contact Hypersensitivity. Eur. J. Immunol..

[14] Torres M.J., Mayorga C., Cornejo-Garcia J.A., Lopez S., Chaves P., Rondon C., Fernandez T., Blanca M. (2008). Monitoring Non-Immediate Allergic Reactions to Iodine Contrast Media. Clin. Exp. Immunol..

[15] Nishio D., Izu K., Kabashima K., Tokura Y. (2007). T Cell Populations Propagating in the Peripheral Blood of Patients with Drug Eruptions. J. Dermatol. Sci..

[16] Beeler A., Zaccaria L., Kawabata T., Gerber B.O., Pichler W.J. (2008). CD69 Upregulation on T Cells as an in Vitro Marker for Delayed-Type Drug Hypersensitivity. Allergy.

[17] Caballero M.L., Quirce S. (2020). Delayed Hypersensitivity Reactions Caused by Drug Excipients: A Literature Review. J. Investig. Allergol. Clin. Immunol..

[18] Zahid S., Mustafa A., Dina A., Sawsan B., Felwa A.M., Mohammed G.E.R., Hasan A. (2019). Nickel Challenge up Regulates CD69 Expression on T Lymphocyte Sub-Sets from Patients with Nickel Induced Contact Dermatitis. Afr. Health Sci..

[19] Takeuchi M., Taguchi M., Sato T., Karasawa K., Sakurai Y., Harimoto K., Ito M. (2017). Association of High-Mobility Group Box-1 with Th Cell-Related Cytokines in the Vitreous of Ocular Sarcoidosis Patients. Investig. Opthalmology Vis. Sci..

[20] Deng N., Chen Q., Guo X., Liu L., Chen S., Wang A., Li R., Huang Y., Ding X., Yu H. (2020). Blockade of CD40L Inhibits Immunogenic Maturation of Lung Dendritic Cells: Implications for the Role of Lung INKT Cells in Mouse Models of Asthma. Mol. Immunol..

[21] Santos A.F., Alpan O., Hoffmann H.-J. (2021). Basophil Activation Test: Mechanisms and Considerations for Use in Clinical Trials and Clinical Practice. Allergy.

[22] Romantowski J., Górska A., Niedoszytko M., Gulen T., Gruchała-Niedoszytko M., Nedoszytko B., Lange M., Brockow K., Arock M., Akin C. (2021). A Challenge for Allergologist: Application of Allergy Diagnostic Methods in Mast Cell Disorders. Int. J. Mol. Sci..

[23] Sánchez-Borges M., Aberer W., Brockow K., Celik G.E., Cernadas J., Greenberger P.A., Masse M.-S., Schrijvers R., Trautmann A. (2019). Controversies in Drug Allergy: Radiographic Contrast Media. J. Allergy Clin. Immunol. Pr..

[24] Zubchenko S., Havrylyuk A., Lomikovska M., Kril I., Chuiko S. (2022). Diagnosis of an Allergic Reaction to Antibiotics in an Patient with Active Human Herpesvirus -4, -6 Type Infection (CLINICAL CASE). Georgian Med. News.

[25] Cornejo-Garcia J.A., Fernandez T.D., Torres M.J., Carballo M., Hernan I., Antunez C., Blanca M., Mayorga C. (2007). Differential Cytokine and Transcription Factor Expression in Patients with Allergic Reactions to Drugs. Allergy.

[26] Posadas S.J., Leyva L., Torres M.J., Rodriguez J.L., Bravo I., Rosal M., Fernandez J., Juarez C., Blanca M. (2000). Subjects with Allergic Reactions to Drugs Show in Vivo Polarized Patterns of Cytokine Expression Depending on the Chronology of the Clinical Reaction. J. Allergy Clin. Immunol..

[27] Tang W., Xie H., Ye Z., Eick-Cost A.A., Scheckelhoff M., Gustin C.E., Bream J.H., Plant E.P. (2023). Post-Vaccination Serum Cytokines Levels Correlate with Breakthrough Influenza Infections. Sci. Rep..

[28] Wang C., Yang S., Duan L., Du X., Tao J., Wang Y., Yang J., Lv Y., Li J., Zhang C. (2022). Adaptive Immune Responses and Cytokine Immune Profiles in Humans Following Prime and Boost Vaccination with the SARS-CoV-2 CoronaVac Vaccine. Virol. J..

[29] Mourmouris P., Tzelves L., Roidi C., Fotsali A. (2021). COVID-19 Transmission: A Rapid Systematic Review of Current Knowledge. Osong Public Health Res. Perspect..

[30] Bruusgaard-Mouritsen M.A., Jensen B.M., Poulsen L.K., Duus Johansen J., Garvey L.H. (2022). Optimizing Investigation of Suspected Allergy to Polyethylene Glycols. J. Allergy Clin. Immunol..

[31] Risma K.A. (2021). COVID-19 MRNA Vaccine Allergy. Curr. Opin. Pediatr..

[32] Johansen J.D., Aalto-Korte K., Agner T., Andersen K.E., Bircher A., Bruze M., Cannavõ A., Giménez-Arnau A., Gonçalo M., Goossens A. (2015). European Society of Contact Dermatitis Guideline for Diagnostic Patch Testing—Recommendations on Best Practice. Contact Dermat..

[33] Heinzerling L., Mari A., Bergmann K.C., Bresciani M., Burbach G., Darsow U., Durham S., Fokkens W., Gjomarkaj M., Haahtela T. (2013). The Skin Prick Test—European Standards. Clin. Transl. Allergy.

[34] Schnyder B., Pichler W.J. (2009). Mechanisms of Drug-Induced Allergy. Mayo Clin. Proc..

[35] Unoa K. (2018). Pathogenic Mechanism and Diagnostic Testing for Drug Allergies. Yakugaku Zasshi.

[36] Wilkinson S.M., Gonçalo M., Aerts O., Badulici S., Dickel H., Gallo R., Garcia-Abujeta J.L., Giménez-Arnau A.M., Hamman C., Hervella M. (2023). The European Baseline Series and Recommended Additions: 2023. Contact Dermat..

[37] Hallab N.J., Caicedo M., Epstein R., McAllister K., Jacobs J.J. (2010). In Vitro Reactivity to Implant Metals Demonstrates a Person-Dependent Association with Both T-Cell and B-Cell Activation. J. Biomed. Mater. Res. A.

[38] Vespa S., Del Biondo P., Simeone P., Cavallucci E., Catitti G., Auciello R., De Bellis D., Altomare I., Pierdomenico L., Canonico B. (2022). Basophil Activation Test with Different Polyethylene Glycols in Patients with Suspected PEG Hypersensitivity Reactions. Int. J. Mol. Sci..

